# Antimicrobial resistance profile of Enterococcus species isolated from intestinal tracts of hospitalized patients in Jimma, Ethiopia

**DOI:** 10.1186/s13104-015-1200-2

**Published:** 2015-06-03

**Authors:** Abdulhakim Abamecha, Beyene Wondafrash, Alemseged Abdissa

**Affiliations:** Department of Biomedical Sciences, Faculty of Public Health and Medical Sciences, Mettu University, P. O. Box: 318, Mettu, Ethiopia; Department of Medical Laboratory Science and Pathology, College of Public Health and Medical Sciences, Jimma University, Jimma, Ethiopia

**Keywords:** Enterococci, Antibiotic resistance, Hospitalized patient, Intestinal tract, Jimma, Ethiopia

## Abstract

**Background:**

Multi-drug-resistant Enterococci colonizing the intestinal tract of hospitalized patients are the major source of infection as well as nosocomial spread. Despite worldwide increasing rate of multidrug resistant Enterococci colonization and infection among hospitalized patients, there is scarcity of data from resource limited setting. The present study aimed at determining the antimicrobial resistance profile of Enterococcus species from intestinal tracts of hospitalized patients in Jimma, Ethiopia.

**Methods:**

The study was conducted among hospitalized patients at Jimma University Specialized Hospital, from January to July 2013. Fecal samples were collected and processed for bacterial isolation and susceptibility testing to antimicrobial agents. Stool samples were inoculated onto enterococcus selective media (Bile Esculin azide agar plate) with and without 6 µg/ml of vancomycin. The isolates were identified to genus and species level by cultural characteristics, Gram’s stain, catalase test, growth in 6.5% NaCl broth, growth at 45°C, motility test and by using API 20 Streptococcus system. Sensitivity testing was done using Kirby–Bauer disk diffusion method. Minimum inhibitory concentrations for vancomycin were determined using E-test strips.

**Result:**

Overall, Enterococci were isolated from 114 (76%) of the study subjects. The isolates were *Enterococcus faecium* (35.1%) followed by *Enterococcusfaecalis* (29.8%), *Enterococcus gallinarum* (17.5%), *Enterococcuscasseliflavus* (8.8%) and *Enterococcusdurans* (8.8%). Among 114 tested Enterococci isolates, 41 (36%) were resistant to ampicillin, 62 (54.4%) to streptomycin and 39 (34.2%) to gentamycin. Other alternative antibiotics to treat mixed nosocomial infection caused by Enterococci also showed high rate of resistance in vitro: ciprofloxacin (50% of resistance), norfloxacin (49.1%), erythromycin (63.2%), tetracycline (64.9%), chloramphenicol (34.2%), and nitrofrantoin (32.4%). Multiple drug resistance was observed among 89.5% of *E. faecium* and *E. faecalis*. Vancomycin resistant Enterococci were observed in 5% of *E. faecium* isolates.

**Conclusion:**

This study reveals high rate of fecal colonization by multidrug-resistant Enterococci and prevalence of vancomycin resistance strains. Thus periodic surveillance of antibacterial susceptibilities is recommended to detect emerging resistance and to prevent the spread of antibacterial-resistant strains.

## Background

Enterococci are Gram-positive cocci that are normal inhabitants of the gastrointestinal tract. However they can also be significant pathogens, causing surgical wound infection, bacteraemia, endocarditis, neonatal sepsis and rarely meningitis [1]. The most common nosocomial infection caused by these organism are urinary tract infection (associated with instrumentation and antimicrobials administration), followed by intra-abdominal and pelvic infection [1, 2]. The relative importance of Enterococcus as a pathogen has increased with the occurrence of high-level resistance to multiple antimicrobial drugs, such as ampicillin, aminoglycosides and vancomycin [3]. The emergence of vancomycin resistance Enterococci (VRE) has alarmed the global infectious diseases community due to few option left for disease management [4]. Besides drug resistant Enterococci colonizing the intestinal tract of hospitalized patients are the major source of infection as well as nosocomial spread [5]. Several trends have been identified in the epidemiology of enterococcal infections: an increasing incidence of enterococcal infections particularly among the severely ill hospitalized patients, an increasing proportion of nosocomial enterococcal infections caused by *Enterococcusfaecium* and an increasing level of resistance to ampicillin, aminoglycosides, and glycopeptides [4–10].

In humans, enterococcal infections may be caused by at least 12 species but most clinical infections are due to either *Enterococcus faecalis* or *E. faecium* [1]. *E. faecalis* is the most common cause (80–90%) followed by *E. faecium* (10–15%). Occasional infections are due to *Enterococcus gallinarum*, *Enterococcus raffinosus*, *Enterococcus casseliflavus*, *Enterococcus avium*, *Enterococcus pseudoavium*, *Enterococcus malodoratus*, *Enterococcus mundtii*, *Enterococcus durans*, and *Enterococcus hirae* [1, 8]. The proportion of isolates of motile Enterococci (*E. gallinarum*, *E. casseliflavus*) remained low, i.e. less than two per cent. It is important to probably recognize the motile Enterococci because they are intrinsically resistant to vancomycin (low level) and inappropriate treatment with vancomycin may contribute to morbidity and mortality [1, 10].

Several studies have documented that enterococcal infections are most commonly caused by the patient’s own commensal flora. Colonization may occur long before or immediately before infection, but either way, it plays a major role in the development of nosocomial infection [5]. Despite the importance of these etiologic agents there is paucity of information regarding antimicrobial resistance of Enterococcus species isolated from intestinal tract of hospitalized patients in the country. Thus, the present study was conducted to determine antimicrobial resistance pattern of fecal Enterococci isolates collected from hospitalized patients in Jimma, Ethiopia.

## Methods

### Study design and area

A cross sectional study was conducted at Jimma University Specialized Hospital (JUSH), which is located 354 km southwest of Addis Ababa, Ethiopia, from January to July 2013. JUSH is a referral hospital in Southwestern region of the Country.

### Study population

One hundred fifty patients, who had at least 10 days of hospital stay at Medical and Surgical wards, were enrolled.

### Specimen collection

A structured questionnaire was used to collect data from the patients after obtaining a written informed consent. Fecal samples were collected in sterile plastic containers which were used for stool collecting and then were transferred to the laboratory. From critically ill patients rectal swabs were collected using sterile cotton swab moistened in sterile normal saline solution at intensive care units. Then, the swabs were immersed in well-labeled Cary-Blair semi-solid medium (Oxoid Ltd, Basingstoke, Hampshire, England) prepared in screw-capped tubes.

### Culture and identification

Stool specimens and rectal swabs were inoculated onto Enterococci selective media [Bile Esculin azide agar plates (Oxoid, Dardilly, France)] with and without 6 µg/ml of vancomycin to recover vancomycin-susceptible isolates and incubated at 37°C for 24 h.

Colonies showing macroscopically morphological differences and whose colony morphology was consistent with that of *Enterococci* [colonies with colourless or grey and surrounded by a black halo (hydrolysis of esculin)] were subcultured and identified as *Enterococci* by additional tests (gram stain, catalase test, 6.5% NaCl test, growth at 45°C and motility test) as recommended by Facklam and Collins [11], Manero and Blanch [12]. Identification of these isolates to species level was performed by API-20 Streptococcus system (bioMe´rieux). For further identification, stock cultures were stored at BHI Broth containing 50% glycerol at −20°C.

### Antibiotic susceptibility testing

Antimicrobial susceptibility studies were performed by disc diffusion (Kirby–Bauer) method according to Clinical Laboratory Standards Institute (CLSI) [13]. The drugs for disc diffusion testing was obtained from Oxoid in the following concentrations: chloramphenicol (CL) (30 μg), gentamicin (HLG) (120 μg), norfloxacin (NOR) (30 μg), ciprofloxacin (CIP) (5 μg), ampicilin (AMP) (10 μg), tetracycline (TE) (30 μg), penicillin (P) (10 IU), erythromycin (ERY) (15 μg), streptomycin (HLS) (300 μg), Nitrofrantoin (FD) (300 μg). Minimum inhibitory concentrations (MICs) for vancomycin were determined using E-test strips (AB Biodisk, Solna, Sweden). *E. faecalis* ATCC 29212 was used as a quality control strain for performing antimicrobial tests.

### Statistical analysis

Data was analyzed using statistical package for social science (SPSS) version 16. Statistical evaluations were carried out at 95% CI and p value <0.05 was considered as significant.

### Ethical considerations

The study was ethically approved by Jimma University Ethical Review Board. Written consent was obtained from patients after explanation of the purpose of the study and procedure of sample collection. Confidentiality of any information related with the patient and their clinical history was maintained.

## Results

### Demographic characteristics

Of the 150 patients, 76 (50.7%) were males and 74 (49.3%) were females resulting in an overall male to female ratio of 1:1. The mean age of the patients was 36 years (range 16–71). Of all 150 study subjects, 92.7% had a history of exposure to one or more antimicrobial agent in the last 2 weeks and 7.3% were without exposure and the average hospital stay was 19.5 days with a range of 10–60.

### Enterococci isolates

Overall, Enterococci were isolated from 114 (76%) of the study subjects, There was no statistically significant difference in the isolation of Enterococci with age (P = 0.432), sex (P = 0.546), hospital duration (0.135) and antibiotic history (P = 0.313) as is shown in Table 1.

**Table 1:** Comparison of demographic characteristics and Enterococcus culture positivity among hospitalized patients in Jimma, Ethiopia

Variables	Culture positive n (%)	Culture negative n (%)	Total (%)	*P*
Age category in years				
16–27	42 (76.4%)	13 (23.6%)	55 (36.7%)	0.432
28–55	62 (73.8%)	22 (26.2%)	84 (56%)	
56 and above	10 (90.9%)	1 (9.1%)	11 (7.3%)	
Sex				
Male	57 (74%)	20 (26%)	77 (51.3%)	0.546
Female	57 (78.1%)	16 (21.9%)	73 (48.7%)	
Hospital duration				
10–30 days	92 (73.6%)	33 (26.4%)	125 (83.3%)	0.135
31 and above	22 (88%)	3 (12%)	25 (16.7%)	
Previous antibiotic treatment				
Yes	103 (74.1%)	36 (25.9%)	139 (92.7%)	0.313
No	10 (90.9%)	1 (9.1%)	11 (7.3%)	

### Species distribution

**Figure 1:**
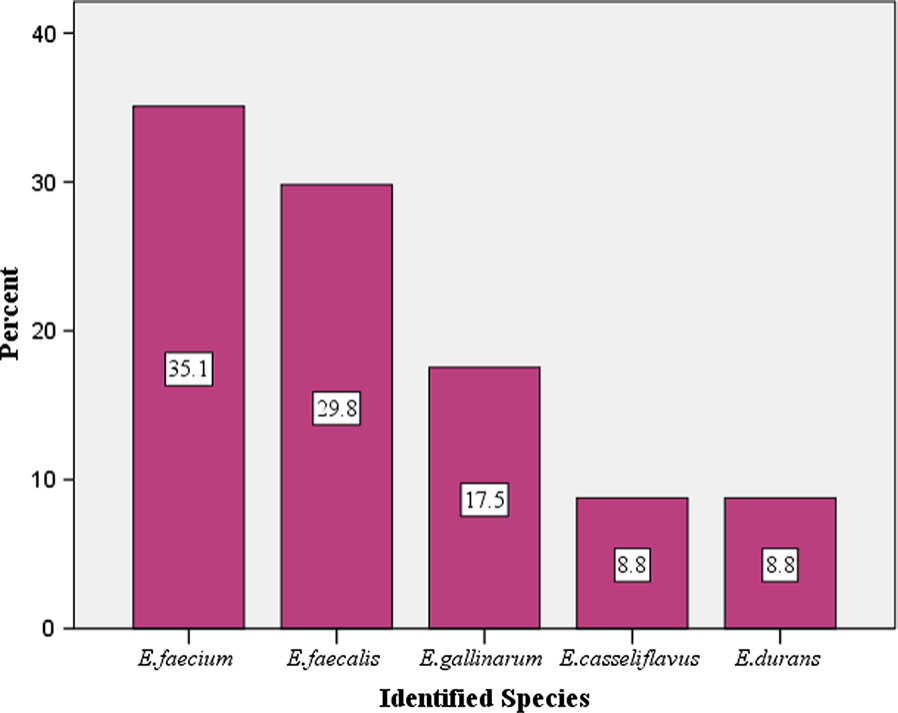
Distribution of Enterococcus species isolated from intestinal tract of hospitalized patients in Jimma, Ethiopia.

The distribution of species is shown in Figure 1. A total of 114 enterococcal isolates were obtained from 150 patients. The commonly enterococcal isolates were *E. faecium* (35.1%) followed by *E. faecalis* (29.8%), *E. gallinarum* (17.5%), *E. casseliflavus* (8.8%) and *E. durans* (8.8%).

### Antimicrobial resistance of Enterococcal isolates

Antimicrobial resistance of isolated Enterococci are summarized in Table 2.

**Table 2:** Antibiotic resistance profile of *Enterococcus species* by Kirby–Bauer disc diffusion method from intestinal tract of hospitalized patients in Jimma, Ethiopia

Antibiotics	Resistant isolates (%)			
	*E. faecalis* (n, 34)	*E. faecium* (n, 40)	Other species^a^ (n, 40)	Total (n, 114)
Ampicillin	5 (14.7)	35 (87.5)	1 (2.5)	41 (36)
Penicillin	34 (100)	40 (100)	11 (27.5)	85 (74.6)
Gentamicin (HLR-GN)	9 (26.5)	30 (75)	1 (2.5)	40 (35.1)
Streptomycin (HLR-ST)	25 (73.5)	36 (90)	1 (2.5)	62 (54.4)
Ciprofloxacin	21 (61.8)	35 (87.5)	1 (2.5)	57 (50)
Norfloxacin	20 (58.8)	34 (85)	2 (5)	56 (49.1)
Erythromycin	25 (73.5)	39 (97.5)	8 (20)	72 (63.2)
Chloramphenicol	25 (73.5)	11 (27.5)	3 (7.5)	39 (34.2)
Tetracycline	32 (94.1)	37 (92.5)	5 (12.5)	74 (64.9)
Nitrofrantoin	17 (50)	18 (45)	2 (5)	37 (32.4)

*HLR-GN* high level resistance to gentamycin, *HLR-ST* high level resistance to streptomycin.

^a^Other species consists of *E. gallinarum* (n, 20), *E. durans* (n, 10) and *E. casseliflavus* (n, 10).

#### β-Lactam resistance

Five of thirty-four (14.71%) *E. faecalis* and 35/40 (87.5%) *E. faecium* were resistant (overall 41/114; 35.96%) to ampicillin. Notably all *E. faecalis* and *E. faecium* isolates were resistant to penicillin. Eleven of fourty (27.5%) occasional Enterococcus species (*E. gallinarum*, *E. durans* and *E. casseliflavus*) were resistant to penicillin.

#### Aminoglycoside resistance

High-level resistance to gentamicin and streptomycin was detected by the high content disk. Gentamycin resistant were observed in 26.5% of *E. faecalis* and 75% of *E.faecium*. Eighteen of thirty-four (52.9%) *E*. *faecalis* and 62.5% *E. faecium* were resistant to streptomycin.

#### Vancomycin-resistant Enterococci

None of the *E. faecalis* isolates tested exhibited resistance to vancomycin while 2 (5%) of *E. faecium* isolates were resistant to vancomycin.

#### Other antimicrobials

Alternative antibiotics to treat infection by Enterococcus also showed high rate of resistance. Resistance to ciprofloxacin was observed in 50% of the isolates, 49.1% to norfloxacin, 63.2% to erythromycin, 34.2% to chloramphenicol, 64.9% to tetracycline and 32.4% to nitrofrantoin.

The drug resistance among the isolates is shown in Table 3. Out of 114 Enterococci isolates 102 (89.5%) were resistant to three antibiotics, 55 (48.2%) were resistant to four antibiotics and 29 (25.4%) were resistant to three antibiotics from different antibiotics classes.

**Table 3:** Multidrug-resistance patterns of *E. faecium and E. faecalis* from intestinal tract of hospitalized patients in Jimma, Ethiopia

No. of antibiotics	Resistance patterns	Number of *E. faecium* with pattern	Number of *E. faecalis* with pattern	Total number of MDR (%)
R3	AMP, HLR-GN, TE	30	5	102 (89.5)
	HLR-GN, TE, FD	16	5	
	TE, FD, CIP	17	13	
	FD, ERY, CL	5	11	
R4	AMP, HLR-GN, TE, FD	16	3	55 (48.2)
	HLR-GN, TE, FD, CIP	16	4	
	TE, FD, ERY, CL	5	11	
R5	AMP, HLR-GN, TE, FD, CIP	16	2	29 (25.4)
	HLR-GN, TE, FD, ERY, CL	5	4	
	TE, FD, ERY, CL, VA	2	0	
R6	HLR-GN, TE, FD, ERY, CL, VA	2 (100)	0	2 (1.75)
R7	AMP, HLR-GN, TE, FD, ERY, CL, VA	2 (100)	0	2 (1.75)

*MDR* non-susceptible to ≥1 agent in ≥3 antimicrobial categories, *R3* resistance to three antibiotics, *R4* resistance to four antibiotics, *R5* resistance to five antibiotics, *R6* resistance to six antibiotics, *R7* resistance to seven antibiotics, *AMP* ampicilin, *HLR-GN* high level resistance to gentamycin, *TE* tetracycline, *FD* Nitrofrantoin, *CIP* ciprofloxacin, *CL* chloramphenicol, *ERY* erythromycin, *VA* vancomycin.

The combined high level aminoglycoside and ampicillin resistance among the Enterococci isolates is shown in Table 4. Resistance among *E. faecium* isolates was higher than *E. faecalis* (85.7 vs. 14.7%).

**Table 4:** Combined high level aminoglycoside and ampicillin resistance of *E. faecium* and *E. faecalis* isolates from intestinal tract of hospitalized patients in Jimma, Ethiopia

Organism (no. of isolates)	Number (%) of combined drug resistant pattern		
	HLR-GN, HLR-ST	AMP	AMP, HLR-GN, HLR-ST
*E. faecium* (40)	30 (75)	35 (87.5)	30 (75)
*E. faecalis* (34)	8 (23.5)	5 (14.7)	5 (14.7)

*AMP* ampicillin, *HLR-GN* high level resistance to gentamycin, *HLR-ST* high level resistance to streptomycin.

## Discussions

The rapid emergence of resistance in Enterococci and the increasing incidence of colonization and infection with VRE have become health care issues that have caused serious concern to physicians and health authorities alike [4]. This study investigated the prevalence of Enterococci and antibacterial resistance patterns of Enterococci isolated from fecal samples of hospitalized patients in wards that have high-risk for VRE colonization in JUSH.

The increase of invasive infections caused by multi-resistant *E. faecium*, however, did not only increase the total burden of nosocomial enterococcal infections, but also resulted in a partial replacement of *E. faecalis* by *E. faecium* as a cause of hospital-associated infections [4]. Several studies showed that an increased proportion of nosocomial enterococcal infections caused by *E.faecium* [14–17]. The isolates obtained in this study were *E. faecium* (35.1%) followed by *E. faecalis* (29.5%) while *E. gallinarium*, *E. casseliflavus*, and *E. durans* accounted for 17.5, 8.8 and 8.8% of the isolates, respectively. This species distribution is comparable to the distribution of enterococcal species in other studies [18, 19]. But it is in disagreement with reports from United States where *E. faecalis* was predominant over *E. fecium* isolates from intestinal tract of hospitalized patients [20].

In our study, the predominant enterococcal isolates was *E.faecium*, which is in concurrence with a recent report from India that described 81% of blood isolates as *E.faecium* [21]. Study in Singapore has also reported an increase in *E. faecium* from 78.9 to 91.8% over a period of 5 years from 2006 to 2010 from clinical cultures [22]. Another study from India has also reported 66% of blood isolates as *E. faecium* [17]. Iwen et al. [14] has also reported an increase in *E. fecium* isolates from 12.9 to 36.3 over a period of 8 years during 1987–1995 from blood cultures.

Although motile Enterococci, including *E. gallinarum* and *E. casseliflavus*, are infrequently isolated from clinical specimens, they have been implicated in a wide variety of invasive infections in humans, especially immunocompromised or chronically ill patients, and sometimes are nosocomially acquired [23, 24]. In our study the prevalence of 17.5% of *E. gallinarum*, 8.8% of *E. casseliflavus* and 8.8% of *E. durans* was significantly higher than in several studies conducted elsewhere [18, 25, 26], although we don’t have explanation for this finding.

The enterococcal isolates possess an intrinsically relative resistance to penicillin and ampicillin. Furthermore, *E. faecium* is less susceptible to β-lactam agents than *E*. *faecalis* because their penicillin-binding proteins (PBPs) have lower affinities for these antibiotics and some strains have plasmid-encoded β-lactamase [1]. In our study, the 14.7% resistance rate to ampicillin in *E. faecalis* isolates was higher than the 0–8.3% resistance rates reported in Kuwait, Hong Kong, and Brazil [5, 18, 27] and comparable to 15% resistant rates reported in Iran [28] and 15.7% in Saudi Arabia [25] and lower than 60.7% reported in Gaza [29] and 66% in India [30]. Resistance rates to ampicillin was observed in 87.5% of *E*. *faecium* isolates which is higher than 31.4% resistance rates reported from Hong Kong [5] and 66.7% in Gaza [29]. However, similar to 87.5% resistance rate reported from Israel among high risk patients [31] and comparable to 82% resistant rate reported from Iran [32]. All *E. faecalis* and *E*. *faecium* isolates were resistant to penicillin which is similar rate of resistance reported from India in clinical isolates [17]. The reason for higher prevalence of β-lactam antibiotic resistance in the examined wards (JUSH) might be because of the set up where chronic cases are prevalent and there is a wider usage of broad spectrum antibiotics relative to other wards.

Aminoglycosides are frequently used in combination with cell-wall-active antibiotics for severe enterococcal infections [4]. Since enterococcal resistance to gentamicin and streptomycin occurs by different mechanisms, it is important to test susceptibility to both agents. Enterococci with high level resistance to streptomycin are susceptible to gentamicin. Gentamicin resistance is a good predictor of resistance to other aminoglycosides except streptomycin [1].

Although high-level aminoglycoside resistance (HLAR) may be regarded as important for severe infections, we determined the high-level resistance in various species to get an idea of the frequency of this kind of resistance in the enterococcal isolates of fecal samples in hospitalized patients. In the present study high-level resistance to gentamicin or streptomycin were observed in 34.2 and 54.4% of Enterococci isolates, respectively. These results are comparable to resistance rate reported from Hong Kong and Japan [5, 33] and higher than resistance rate reported from Kuwait and Saudi Arabia [18, 25]. When high-level resistance to gentamicin and streptomycin occurs in the same strain, it means that, with few exceptions, there is no reliable bactericidal regimen [1]. In our study, *E. faecalis* and *E. faecium* showed resistance to as many as nine drugs. Concomitant resistance of high level aminoglycoside resistance (HLAR) strains to the β-lactam antibiotic (ampicillin) was quite higher (14.7% of *E. faecalis* and 85.7% of *E. faecium* strains) (Table 4). This finding is a cause of concern, because the synergistic activity of the combination of β-lactam antibiotics with HLAR in the treatment of enterococcal infections is totally abolished. In such instances, controlling the spread of these organisms becomes of paramount importance.

Because of high prevalence of concomitant resistance, attempts had been made to look for alternative antibiotics in different studies. Fluroquinolones have been among the dominant class of antimicrobial agents in the last decade and are widely used for nosocomial infections empirically [34]. In our study 50% of Enterococci isolate were resistant to ciprofloxacin and 49.1% of the isolates were resistant to norfloxacin. Other alternative antibiotics to treat infection by Enterococcus also showed high rates of resistance in vitro: erythromycin (63.2% of resistance), tetracycline (64.9%), chloramphenicol (34.2%), and nitrofrantoin (32.4%). This widely used class of antimicrobial agent for empirical treatment of mixed nosocomial infections caused by Enterococci could not be effective in our setting because of high rates of resistance according to the present study.

The emergence of VRE is also due to the inappropriate use of cephalosporin as well as poor hospital infection control measures [1, 35]. As our results showed 2 out of 40 *E. faecium* strains (5%) were vancomycin resistant which is comparable with 4% VRE strains report from Egypt [36] and 6.2% from Iran [37]. Lower than 10.2% report from South Africa [38], 12% report from Korea [39] and 34.8% report from Turkey [40]. The possible reason for the emergence of VRE in studied hospital (JUSH) may possibly be antibiotic selective pressure because the patients in the studied units (medical and surgical ward) had long duration of hospital stay and high rate of antibiotics treatment relative to the other wards which are the most frequently reported risk factor for multi-resistance Enterococci colonization and infection.

## Conclusions

The prevalence of VRE in faeces of hospitalized patients at JUSH was 5%. High percentage of multi-drug resistance was also observed in the majority of the isolates. Overall, multiple drug resistance was observed among 89.5% of *E. faecium* and *E. faecalis*. The emergence of VRE (5%) and the high rate of fecal colonization by multi-resistant Enterococci in this study call for periodic surveillance of antibacterial susceptibilities to detect emerging resistance and prevent the spread of antibacterial-resistant strains.
